# 2-Chloro-1-(3,3-dimethyl-2,6-di­phenyl­piperidin-1-yl)ethanone

**DOI:** 10.1107/S1600536813022289

**Published:** 2013-08-14

**Authors:** K. Prathebha, B. K. Revathi, G. Usha, S. Ponnuswamy, S. Abdul Basheer

**Affiliations:** a PG and Research Department of Physics, Queen Mary’s College, Chennai-4, Tamilnadu, India; b Department of Chemistry, Government Arts College (Autonomous), Coimbatore 641 018, Tamilnadu, India

## Abstract

In the title compound, C_21_H_24_ClNO, the piperidine ring adopts a chair conformation. The two phenyl rings are inclined to one another by 20.7 (1)°, and are inclined to the mean plane of the four planar atoms of the piperidine ring by 87.64 (10) and 70.8 (1)°. The mol­ecular structure features short intra­molecular C—H⋯Cl and C—H⋯O contacts. In the crystal, there are no significant inter­molecular inter­actions present.

## Related literature

For the synthesis of the title compound, see: Venkatraj *et al.* (2008 ▶). For the biological activity of piperdine derivatives, see: Ramalingan *et al.* (2004 ▶), We­intraub *et al.* (2003 ▶); Ramachandran *et al.* (2011 ▶). For a related structure, see: Aridoss *et al.* (2011 ▶). For puckering parameters, see: Cremer & Pople (1975 ▶).
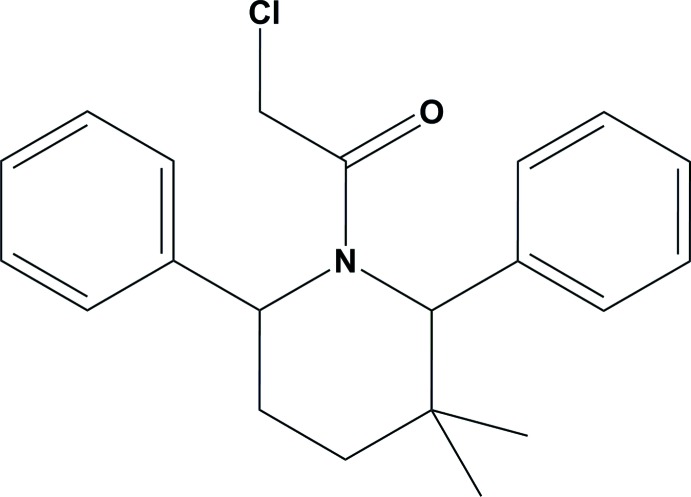


## Experimental

### 

#### Crystal data

C_21_H_24_ClNO
*M* *_r_* = 341.86
Triclinic, 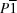

*a* = 7.5488 (6) Å
*b* = 9.9706 (7) Å
*c* = 12.9887 (10) Å
α = 106.783 (4)°
β = 93.022 (4)°
γ = 102.347 (4)°
*V* = 907.45 (12) Å^3^
*Z* = 2
Mo *K*α radiation
μ = 0.22 mm^−1^
*T* = 293 K
0.22 × 0.20 × 0.20 mm

#### Data collection

Bruker Kappa APEXII CCD diffractometer
Absorption correction: multi-scan (*SADABS*; Bruker, 2004 ▶) *T*_min_ = 0.953, *T*_max_ = 0.958
13736 measured reflections
3806 independent reflections
3169 reflections with *I* > 2σ(*I*)
*R*_int_ = 0.026

#### Refinement

*R*[*F* ^2^ > 2σ(*F* ^2^)] = 0.046
*wR*(*F* ^2^) = 0.131
*S* = 1.02
3806 reflections
219 parameters
H-atom parameters constrained
Δρ_max_ = 0.26 e Å^−3^
Δρ_min_ = −0.38 e Å^−3^

### 

Data collection: *APEX2* (Bruker, 2004 ▶); cell refinement: *SAINT* (Bruker, 2004 ▶); data reduction: *SAINT* and *XPREP* (Bruker, 2004 ▶); program(s) used to solve structure: *SHELXS97* (Sheldrick, 2008 ▶); program(s) used to refine structure: *SHELXL97* (Sheldrick, 2008 ▶); molecular graphics: *ORTEP-3 for Windows* (Farrugia, 2012 ▶); software used to prepare material for publication: *SHELXL97* and *PLATON* (Spek, 2009 ▶).

## Supplementary Material

Crystal structure: contains datablock(s) I, New_Global_Publ_Block. DOI: 10.1107/S1600536813022289/su2628sup1.cif

Structure factors: contains datablock(s) I. DOI: 10.1107/S1600536813022289/su2628Isup2.hkl

Supplementary material file. DOI: 10.1107/S1600536813022289/su2628Isup3.cml

Additional supplementary materials:  crystallographic information; 3D view; checkCIF report

## Figures and Tables

**Table 1:** Hydrogen-bond geometry (Å, °)

*D*—H⋯*A*	*D*—H	H⋯*A*	*D*⋯*A*	*D*—H⋯*A*
C7—H7⋯Cl1	0.98	2.68	3.3736 (16)	128
C13—H13⋯O1	0.98	2.27	2.732 (2)	108

**Fig. 1.**
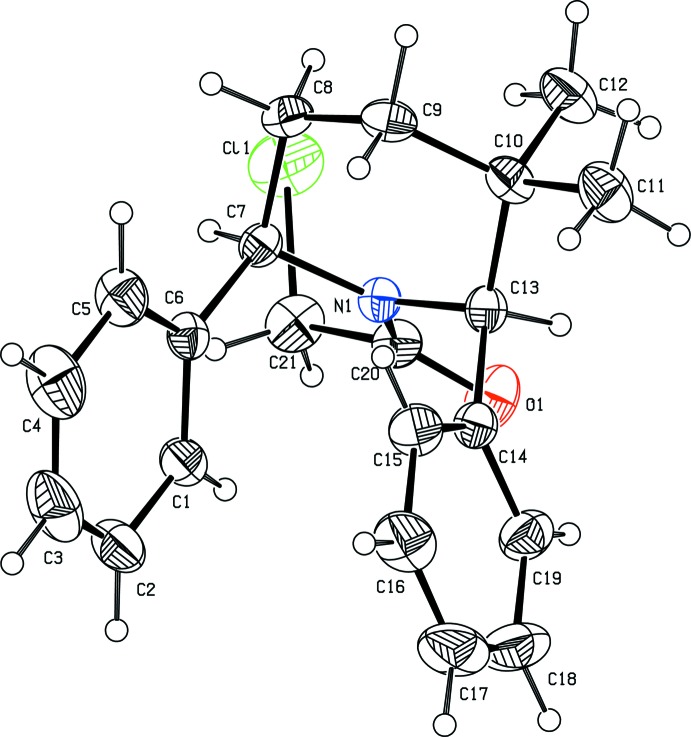
The molecular structure of the title molecule, with displacement ellipsoids drawn at the 30% probability level.

**Fig. 2.**
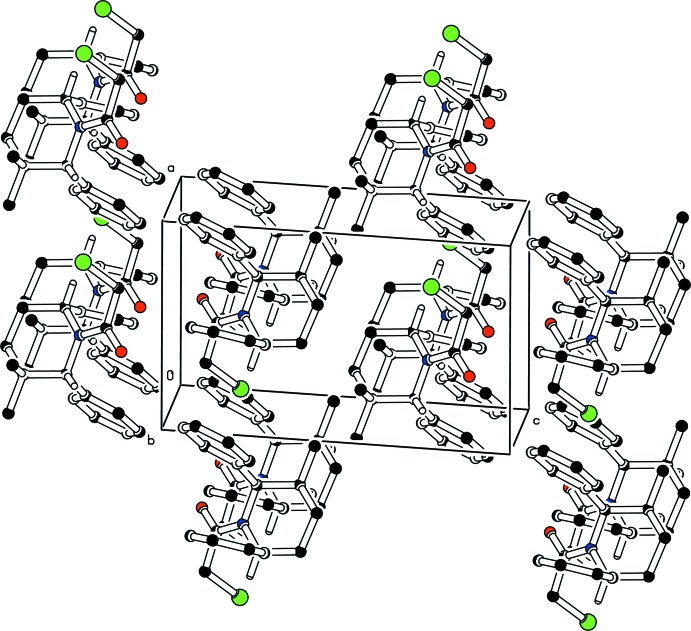
The crystal packing of the title compound, viewed along the b axis. The dashed lines indicate the short intramolecular C-H···O and C-H···Cl contacts (see Table 1 for details).

## supplementary crystallographic information

### 1. Comment

The piperidine sub-structure is a ubiquitous structural feature of many alkaloids, natural products and drug candidates (Weintraub *et al.*, 2003). The motivation for biological trials arises as piperidine derivatives are an important class of heterocyclic compounds with potent pharmacological and biological activities (Ramalingan *et al.*, 2004; Ramachandran *et al.*, 2011). We report herein on the synthesis and crystal structure of a new piperidine derivative.

In the title molecule, Fig. 1, the phenyl rings are attached to the piperidine ring in the symmetric position through bonds C6—C7 [1.5252( ) Å] and C13—C14 [1.523( ) Å]. These bond distances are comparable with those in a related structure (Aridoss *et al.*, 2011). The two phenyl rings (A = C1-C6 and B = C14-C19) are inclined to one another by 20.7 (1) °. The sum of the bond angles around the N atom of the piperidine ring (360 °) shows *sp*^3^ hybridization. The piperidine ring (C7-C10/C13/N1) adopts a chair conformation with puckering parameters (Cremer & Pople, 1975) of Q(2) = 0.0311 (16) Å, φ(2) = 135 (3) ° Q(3) = 0.5222 (16) Å with Puckering Amplitude (Q) = 0.5231 (16) Å, θ = 3.42 (18) °, π = 135 (3) °. The two phenyl rings (A and B) are inclined to the mean plane of the four planar atoms (N1/C13/C9/C8) of piperidine ring by 87.64 (10) and 70.8 (1) °, respectively.

The molecule is stabilized by short intramolecular C—H···Cl and C—H···O contacts (Table 1).

In the crystal, the molecules stack along the c axis direction without any specific interactions (Fig. 2).

### 2. Experimental

The title compound was synthesized according to the published procedure (Venkatraj *et al.*, 2008). A mixture of piperidine (5 mmol), chloroacetylchloride (20 mmol) and triethylamine (20 mmol) in anhydrous benzene (20 ml) was stirred at rt for 7 h. The precipitated ammonium salt was washed with water (4 × 10 ml) and the benzene solution was dried and concentrated. The pasty mass was purified by crystallization from ethanol giving colourless block-like crystals [M.p. 377-379 K].

### 3. Refinement

H atoms were positioned geometrically and treated as riding atoms: C—H = 0.93 - 0.98 Å with *U*_iso_(H) = 1.5U_eq_(C-methyl) and = 1.2U_eq_(N,C) for other H atoms.

### Crystal data

**Table:**

C_21_H_24_ClNO	*Z* = 2
*M**_r_* = 341.86	*F*(000) = 364
Triclinic, *P*1	*D*_x_ = 1.251 Mg m^−^^3^
Hall symbol: -P 1	Mo *K*α radiation, λ = 0.71073 Å
*a* = 7.5488 (6) Å	Cell parameters from 3806 reflections
*b* = 9.9706 (7) Å	θ = 1.7–26.7°
*c* = 12.9887 (10) Å	µ = 0.22 mm^−^^1^
α = 106.783 (4)°	*T* = 293 K
β = 93.022 (4)°	Block, colourless
γ = 102.347 (4)°	0.22 × 0.20 × 0.20 mm
*V* = 907.45 (12) Å^3^	

### Data collection

**Table:**

Bruker Kappa APEXII CCD diffractometer	3806 independent reflections
Radiation source: fine-focus sealed tube	3169 reflections with *I* > 2σ(*I*)
Graphite monochromator	*R*_int_ = 0.026
ω and φ scan	θ_max_ = 26.7°, θ_min_ = 1.7°
Absorption correction: multi-scan (*SADABS*; Bruker, 2004)	*h* = −9→9
*T*_min_ = 0.953, *T*_max_ = 0.958	*k* = −12→12
13736 measured reflections	*l* = −15→16

### Refinement

**Table:**

Refinement on *F*^2^	Primary atom site location: structure-invariant direct methods
Least-squares matrix: full	Secondary atom site location: difference Fourier map
*R*[*F*^2^ > 2σ(*F*^2^)] = 0.046	Hydrogen site location: inferred from neighbouring sites
*wR*(*F*^2^) = 0.131	H-atom parameters constrained
*S* = 1.02	*w* = 1/[σ^2^(*F*_o_^2^) + (0.068*P*)^2^ + 0.2438*P*] where *P* = (*F*_o_^2^ + 2*F*_c_^2^)/3
3806 reflections	(Δ/σ)_max_ < 0.001
219 parameters	Δρ_max_ = 0.26 e Å^−^^3^
0 restraints	Δρ_min_ = −0.38 e Å^−^^3^

### Special details

**Table:**

Geometry. All e.s.d.'s (except the e.s.d. in the dihedral angle between two l.s. planes) are estimated using the full covariance matrix. The cell e.s.d.'s are taken into account individually in the estimation of e.s.d.'s in distances, angles and torsion angles; correlations between e.s.d.'s in cell parameters are only used when they are defined by crystal symmetry. An approximate (isotropic) treatment of cell e.s.d.'s is used for estimating e.s.d.'s involving l.s. planes.
Refinement. Refinement of *F*^2^ against ALL reflections. The weighted *R*-factor *wR* and goodness of fit *S* are based on *F*^2^, conventional *R*-factors *R* are based on *F*, with *F* set to zero for negative *F*^2^. The threshold expression of *F*^2^ > σ(*F*^2^) is used only for calculating *R*-factors(gt) *etc*. and is not relevant to the choice of reflections for refinement. *R*-factors based on *F*^2^ are statistically about twice as large as those based on *F*, and *R*- factors based on ALL data will be even larger.

### Fractional atomic coordinates and isotropic or equivalent isotropic displacement parameters (Å^2^)

**Table:**

	*x*	*y*	*z*	*U*_iso_*/*U*_eq_	
C1	0.5516 (3)	0.48807 (17)	0.32195 (15)	0.0580 (4)	
H1	0.5332	0.5009	0.3941	0.070*	
C2	0.5903 (3)	0.6061 (2)	0.2849 (2)	0.0786 (6)	
H2	0.5981	0.6977	0.3322	0.094*	
C3	0.6172 (3)	0.5900 (2)	0.1796 (2)	0.0799 (7)	
H3	0.6462	0.6701	0.1553	0.096*	
C4	0.6009 (3)	0.4528 (2)	0.10924 (17)	0.0667 (5)	
H4	0.6164	0.4405	0.0368	0.080*	
C5	0.5619 (2)	0.33496 (17)	0.14573 (13)	0.0482 (4)	
H5	0.5503	0.2432	0.0976	0.058*	
C6	0.53966 (19)	0.35088 (14)	0.25340 (11)	0.0391 (3)	
C7	0.49478 (19)	0.21737 (14)	0.29103 (11)	0.0368 (3)	
H7	0.3659	0.1703	0.2648	0.044*	
C8	0.5168 (2)	0.24431 (17)	0.41304 (12)	0.0457 (3)	
H8A	0.4581	0.3206	0.4471	0.055*	
H8B	0.4553	0.1576	0.4284	0.055*	
C9	0.7153 (2)	0.28648 (18)	0.46186 (11)	0.0490 (4)	
H9A	0.7209	0.2955	0.5384	0.059*	
H9B	0.7728	0.3799	0.4552	0.059*	
C10	0.8214 (2)	0.17659 (16)	0.40704 (12)	0.0455 (3)	
C11	0.7480 (3)	0.0335 (2)	0.43042 (16)	0.0647 (5)	
H11A	0.8242	−0.0315	0.4036	0.097*	
H11B	0.6254	−0.0088	0.3951	0.097*	
H11C	0.7486	0.0514	0.5071	0.097*	
C12	1.0240 (3)	0.2299 (2)	0.45228 (16)	0.0638 (5)	
H12A	1.0714	0.3230	0.4439	0.096*	
H12B	1.0901	0.1630	0.4136	0.096*	
H12C	1.0374	0.2371	0.5277	0.096*	
C13	0.79807 (19)	0.14287 (14)	0.28205 (11)	0.0386 (3)	
H13	0.8313	0.0504	0.2540	0.046*	
C14	0.91734 (19)	0.24389 (16)	0.23129 (12)	0.0424 (3)	
C15	0.9668 (2)	0.39355 (18)	0.27324 (15)	0.0541 (4)	
H15	0.9322	0.4386	0.3397	0.065*	
C16	1.0665 (3)	0.4759 (2)	0.21753 (19)	0.0718 (6)	
H16	1.0980	0.5759	0.2464	0.086*	
C17	1.1192 (3)	0.4109 (3)	0.1199 (2)	0.0850 (7)	
H17	1.1846	0.4669	0.0820	0.102*	
C18	1.0757 (3)	0.2639 (3)	0.07813 (18)	0.0800 (7)	
H18	1.1131	0.2197	0.0124	0.096*	
C19	0.9761 (2)	0.1810 (2)	0.13376 (14)	0.0572 (4)	
H19	0.9479	0.0810	0.1051	0.069*	
C20	0.5286 (2)	−0.00943 (15)	0.15914 (12)	0.0437 (3)	
C21	0.3245 (2)	−0.05018 (19)	0.12339 (14)	0.0570 (4)	
H21A	0.2973	−0.1266	0.0546	0.068*	
H21B	0.2871	0.0326	0.1129	0.068*	
N1	0.60093 (15)	0.11277 (11)	0.24134 (9)	0.0357 (3)	
O1	0.62001 (18)	−0.08988 (13)	0.11198 (11)	0.0661 (4)	
Cl1	0.19890 (8)	−0.10917 (6)	0.22079 (5)	0.0865 (2)	

### Atomic displacement parameters (Å^2^)

**Table:**

	*U*^11^	*U*^22^	*U*^33^	*U*^12^	*U*^13^	*U*^23^
C1	0.0763 (12)	0.0397 (8)	0.0540 (10)	0.0194 (8)	0.0015 (8)	0.0055 (7)
C2	0.1003 (17)	0.0380 (9)	0.0930 (16)	0.0173 (9)	−0.0070 (12)	0.0162 (9)
C3	0.0786 (14)	0.0593 (11)	0.1160 (19)	0.0119 (10)	−0.0009 (13)	0.0540 (12)
C4	0.0679 (12)	0.0803 (13)	0.0706 (12)	0.0237 (10)	0.0102 (9)	0.0470 (10)
C5	0.0533 (9)	0.0506 (8)	0.0447 (8)	0.0168 (7)	0.0043 (7)	0.0178 (7)
C6	0.0399 (7)	0.0361 (7)	0.0401 (7)	0.0114 (6)	0.0008 (6)	0.0089 (5)
C7	0.0368 (7)	0.0348 (6)	0.0361 (7)	0.0092 (5)	0.0055 (5)	0.0062 (5)
C8	0.0507 (9)	0.0489 (8)	0.0375 (7)	0.0123 (7)	0.0134 (6)	0.0118 (6)
C9	0.0589 (10)	0.0537 (9)	0.0302 (7)	0.0081 (7)	0.0029 (6)	0.0112 (6)
C10	0.0470 (8)	0.0481 (8)	0.0422 (8)	0.0078 (6)	−0.0021 (6)	0.0189 (6)
C11	0.0736 (12)	0.0623 (10)	0.0682 (11)	0.0131 (9)	0.0026 (9)	0.0388 (9)
C12	0.0537 (10)	0.0718 (11)	0.0631 (11)	0.0091 (9)	−0.0133 (8)	0.0242 (9)
C13	0.0382 (7)	0.0345 (6)	0.0423 (7)	0.0101 (5)	0.0032 (6)	0.0099 (5)
C14	0.0326 (7)	0.0518 (8)	0.0452 (8)	0.0102 (6)	0.0038 (6)	0.0186 (6)
C15	0.0470 (9)	0.0522 (9)	0.0629 (10)	0.0047 (7)	0.0092 (7)	0.0225 (8)
C16	0.0549 (11)	0.0711 (12)	0.0933 (15)	−0.0019 (9)	0.0085 (10)	0.0439 (11)
C17	0.0565 (12)	0.121 (2)	0.0891 (16)	−0.0019 (12)	0.0158 (11)	0.0653 (15)
C18	0.0545 (11)	0.126 (2)	0.0589 (11)	0.0107 (12)	0.0201 (9)	0.0330 (12)
C19	0.0432 (9)	0.0762 (11)	0.0496 (9)	0.0142 (8)	0.0084 (7)	0.0149 (8)
C20	0.0495 (8)	0.0341 (7)	0.0413 (7)	0.0065 (6)	0.0041 (6)	0.0049 (6)
C21	0.0521 (10)	0.0507 (9)	0.0508 (9)	0.0007 (7)	−0.0027 (7)	−0.0011 (7)
N1	0.0376 (6)	0.0304 (5)	0.0363 (6)	0.0075 (4)	0.0030 (5)	0.0067 (4)
O1	0.0637 (8)	0.0485 (6)	0.0685 (8)	0.0174 (6)	0.0048 (6)	−0.0115 (6)
Cl1	0.0704 (4)	0.0732 (4)	0.1052 (5)	−0.0123 (3)	0.0181 (3)	0.0310 (3)

### Geometric parameters (Å, º)

**Table:**

C1—C6	1.381 (2)	C11—H11B	0.9600
C1—C2	1.377 (3)	C11—H11C	0.9600
C1—H1	0.9300	C12—H12A	0.9600
C2—C3	1.361 (3)	C12—H12B	0.9600
C2—H2	0.9300	C12—H12C	0.9600
C3—C4	1.384 (3)	C13—N1	1.4896 (17)
C3—H3	0.9300	C13—C14	1.523 (2)
C4—C5	1.371 (2)	C13—H13	0.9800
C4—H4	0.9300	C14—C19	1.384 (2)
C5—C6	1.385 (2)	C14—C15	1.391 (2)
C5—H5	0.9300	C15—C16	1.378 (3)
C6—C7	1.5252 (19)	C15—H15	0.9300
C7—N1	1.4729 (17)	C16—C17	1.369 (4)
C7—C8	1.5228 (19)	C16—H16	0.9300
C7—H7	0.9800	C17—C18	1.367 (4)
C8—C9	1.517 (2)	C17—H17	0.9300
C8—H8A	0.9700	C18—C19	1.381 (3)
C8—H8B	0.9700	C18—H18	0.9300
C9—C10	1.528 (2)	C19—H19	0.9300
C9—H9A	0.9700	C20—O1	1.2214 (19)
C9—H9B	0.9700	C20—N1	1.3510 (17)
C10—C12	1.531 (2)	C20—C21	1.518 (2)
C10—C11	1.538 (2)	C21—Cl1	1.779 (2)
C10—C13	1.552 (2)	C21—H21A	0.9700
C11—H11A	0.9600	C21—H21B	0.9700
			
C6—C1—C2	120.83 (18)	C10—C11—H11C	109.5
C6—C1—H1	119.6	H11A—C11—H11C	109.5
C2—C1—H1	119.6	H11B—C11—H11C	109.5
C3—C2—C1	120.67 (18)	C10—C12—H12A	109.5
C3—C2—H2	119.7	C10—C12—H12B	109.5
C1—C2—H2	119.7	H12A—C12—H12B	109.5
C2—C3—C4	119.18 (17)	C10—C12—H12C	109.5
C2—C3—H3	120.4	H12A—C12—H12C	109.5
C4—C3—H3	120.4	H12B—C12—H12C	109.5
C5—C4—C3	120.35 (19)	N1—C13—C14	111.88 (11)
C5—C4—H4	119.8	N1—C13—C10	109.68 (11)
C3—C4—H4	119.8	C14—C13—C10	119.26 (12)
C4—C5—C6	120.80 (16)	N1—C13—H13	104.9
C4—C5—H5	119.6	C14—C13—H13	104.9
C6—C5—H5	119.6	C10—C13—H13	104.9
C1—C6—C5	118.13 (14)	C19—C14—C15	117.72 (16)
C1—C6—C7	122.40 (14)	C19—C14—C13	116.89 (14)
C5—C6—C7	119.42 (12)	C15—C14—C13	125.35 (14)
N1—C7—C8	108.53 (11)	C16—C15—C14	120.80 (18)
N1—C7—C6	111.44 (11)	C16—C15—H15	119.6
C8—C7—C6	116.13 (11)	C14—C15—H15	119.6
N1—C7—H7	106.7	C15—C16—C17	120.2 (2)
C8—C7—H7	106.7	C15—C16—H16	119.9
C6—C7—H7	106.7	C17—C16—H16	119.9
C9—C8—C7	112.77 (12)	C18—C17—C16	120.09 (19)
C9—C8—H8A	109.0	C18—C17—H17	120.0
C7—C8—H8A	109.0	C16—C17—H17	120.0
C9—C8—H8B	109.0	C17—C18—C19	119.9 (2)
C7—C8—H8B	109.0	C17—C18—H18	120.1
H8A—C8—H8B	107.8	C19—C18—H18	120.1
C8—C9—C10	112.41 (12)	C14—C19—C18	121.28 (19)
C8—C9—H9A	109.1	C14—C19—H19	119.4
C10—C9—H9A	109.1	C18—C19—H19	119.4
C8—C9—H9B	109.1	O1—C20—N1	123.03 (14)
C10—C9—H9B	109.1	O1—C20—C21	117.96 (13)
H9A—C9—H9B	107.9	N1—C20—C21	119.00 (13)
C12—C10—C9	110.51 (14)	C20—C21—Cl1	111.42 (12)
C12—C10—C11	107.52 (13)	C20—C21—H21A	109.3
C9—C10—C11	109.57 (14)	Cl1—C21—H21A	109.3
C12—C10—C13	110.43 (14)	C20—C21—H21B	109.3
C9—C10—C13	111.76 (11)	Cl1—C21—H21B	109.3
C11—C10—C13	106.88 (13)	H21A—C21—H21B	108.0
C10—C11—H11A	109.5	C20—N1—C7	123.13 (12)
C10—C11—H11B	109.5	C20—N1—C13	117.91 (11)
H11A—C11—H11B	109.5	C7—N1—C13	118.95 (10)
			
C6—C1—C2—C3	0.2 (3)	C10—C13—C14—C19	142.88 (14)
C1—C2—C3—C4	1.5 (4)	N1—C13—C14—C15	90.66 (17)
C2—C3—C4—C5	−1.4 (3)	C10—C13—C14—C15	−39.2 (2)
C3—C4—C5—C6	−0.5 (3)	C19—C14—C15—C16	1.8 (2)
C2—C1—C6—C5	−2.0 (3)	C13—C14—C15—C16	−176.05 (15)
C2—C1—C6—C7	−179.24 (17)	C14—C15—C16—C17	−0.4 (3)
C4—C5—C6—C1	2.2 (2)	C15—C16—C17—C18	−1.1 (3)
C4—C5—C6—C7	179.49 (15)	C16—C17—C18—C19	1.0 (3)
C1—C6—C7—N1	−141.81 (15)	C15—C14—C19—C18	−1.9 (2)
C5—C6—C7—N1	41.02 (17)	C13—C14—C19—C18	176.13 (16)
C1—C6—C7—C8	−16.9 (2)	C17—C18—C19—C14	0.6 (3)
C5—C6—C7—C8	165.97 (13)	O1—C20—C21—Cl1	107.89 (16)
N1—C7—C8—C9	52.72 (16)	N1—C20—C21—Cl1	−71.80 (17)
C6—C7—C8—C9	−73.70 (16)	O1—C20—N1—C7	173.64 (14)
C7—C8—C9—C10	−54.77 (17)	C21—C20—N1—C7	−6.7 (2)
C8—C9—C10—C12	175.17 (13)	O1—C20—N1—C13	−5.6 (2)
C8—C9—C10—C11	−66.52 (16)	C21—C20—N1—C13	174.10 (13)
C8—C9—C10—C13	51.76 (17)	C8—C7—N1—C20	126.99 (14)
C12—C10—C13—N1	−171.49 (12)	C6—C7—N1—C20	−103.91 (15)
C9—C10—C13—N1	−48.04 (15)	C8—C7—N1—C13	−53.80 (15)
C11—C10—C13—N1	71.84 (15)	C6—C7—N1—C13	75.29 (14)
C12—C10—C13—C14	−40.64 (18)	C14—C13—N1—C20	96.58 (15)
C9—C10—C13—C14	82.81 (16)	C10—C13—N1—C20	−128.75 (13)
C11—C10—C13—C14	−157.31 (13)	C14—C13—N1—C7	−82.66 (14)
N1—C13—C14—C19	−87.25 (15)	C10—C13—N1—C7	52.00 (15)

### Hydrogen-bond geometry (Å, º)

**Table:**

*D*—H···*A*	*D*—H	H···*A*	*D*···*A*	*D*—H···*A*
C7—H7···Cl1	0.98	2.68	3.3736 (16)	128
C13—H13···O1	0.98	2.27	2.732 (2)	108
